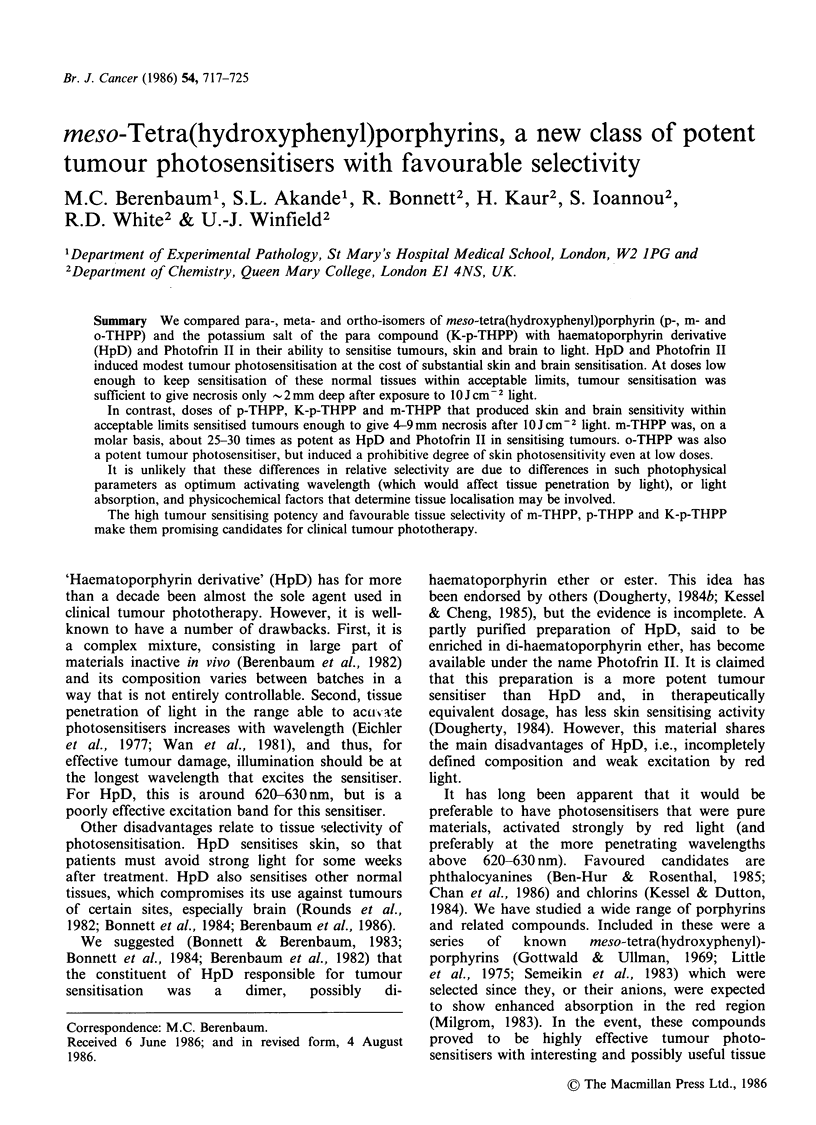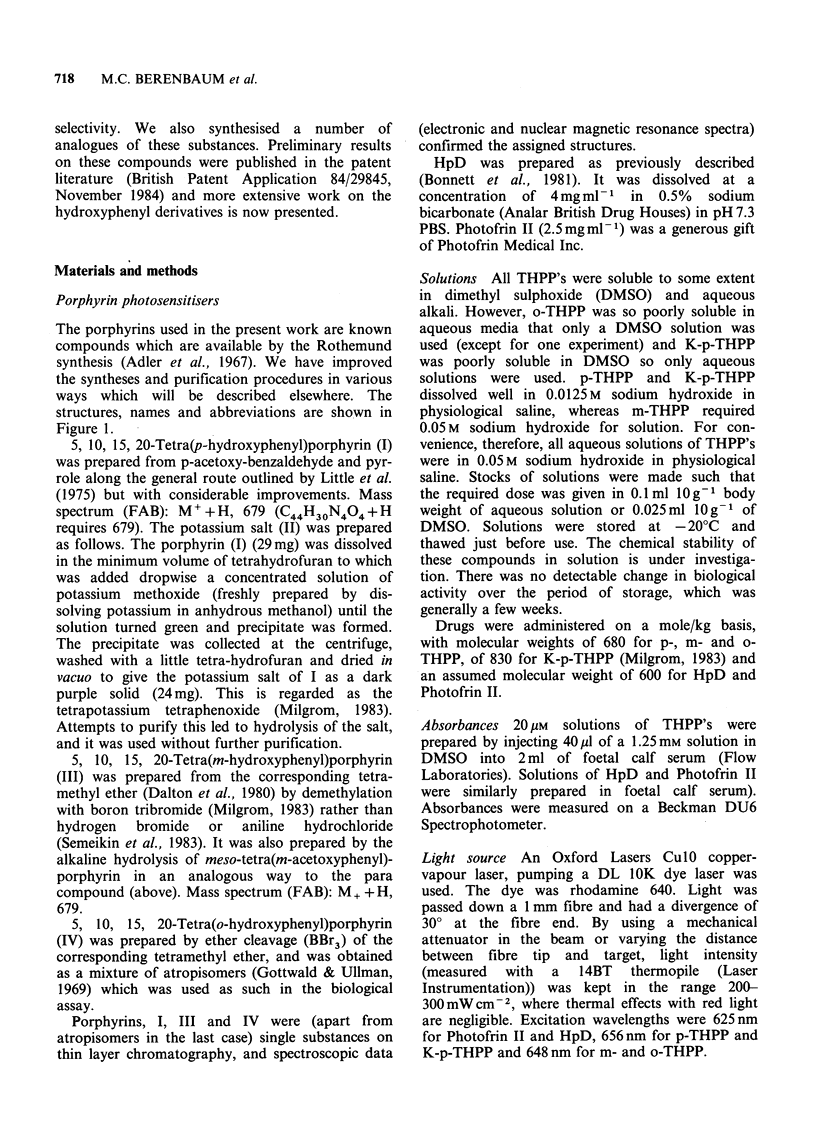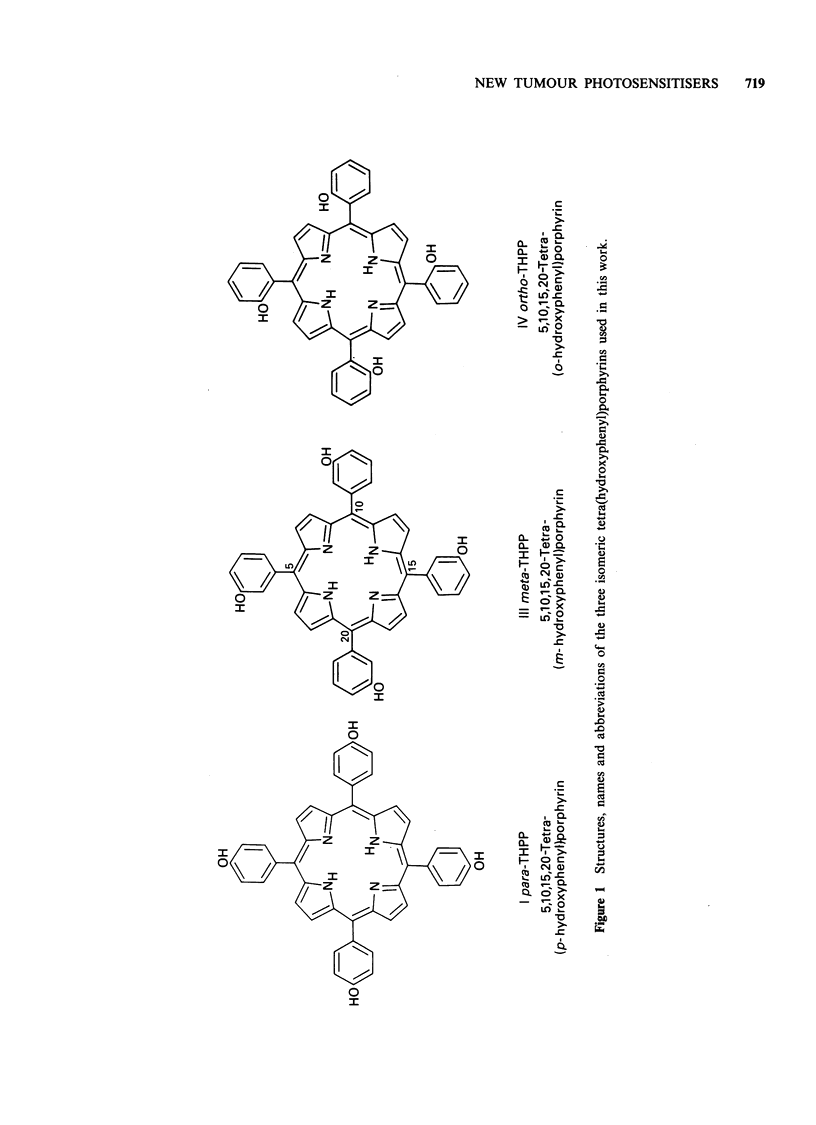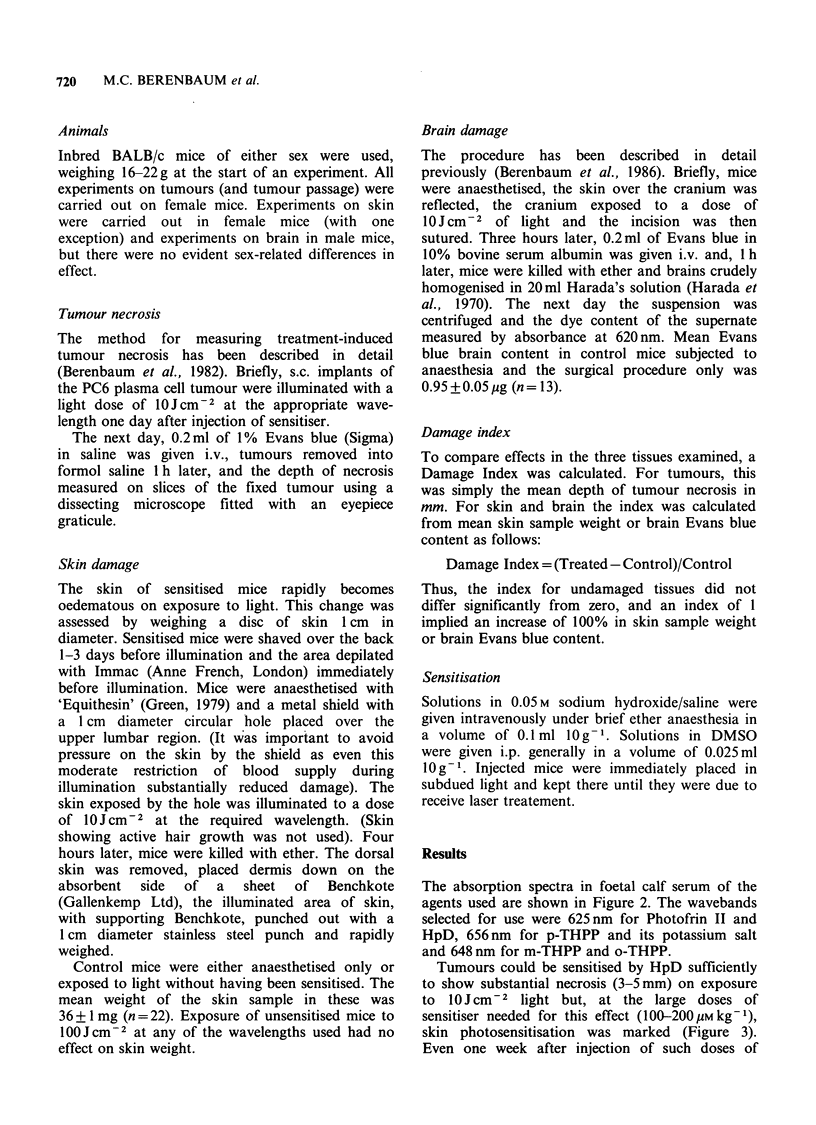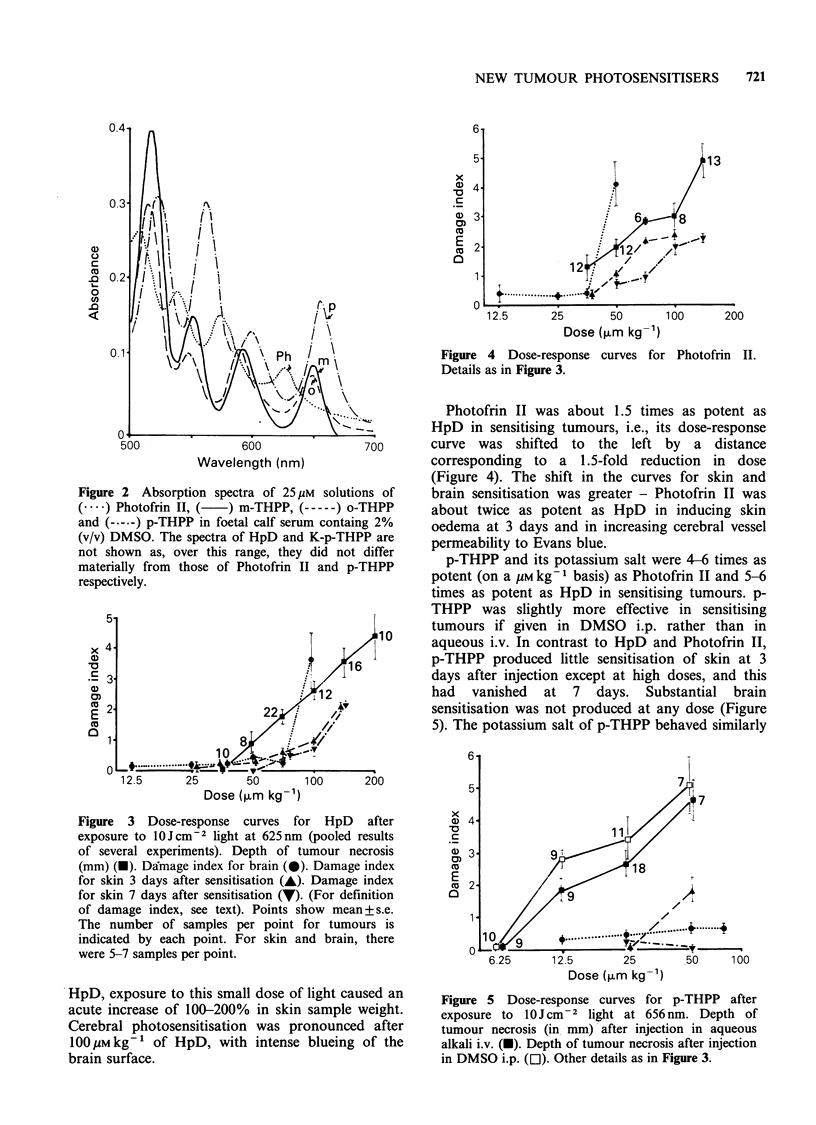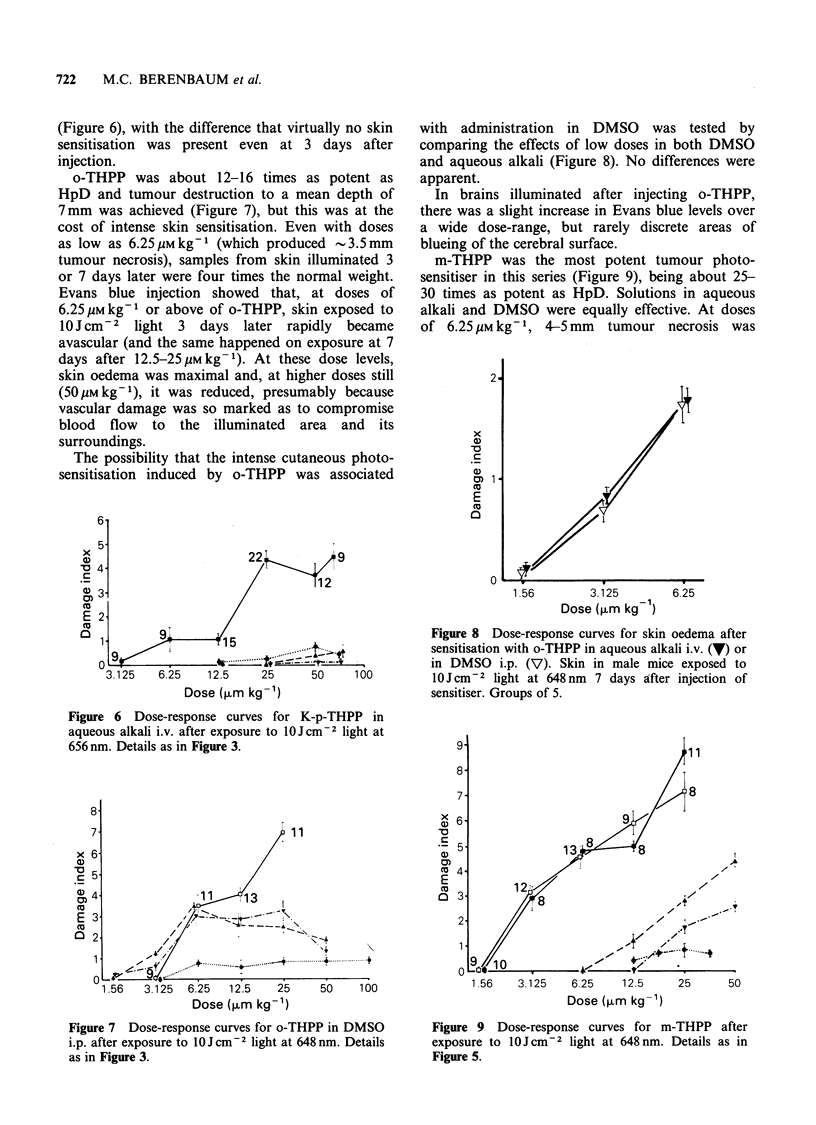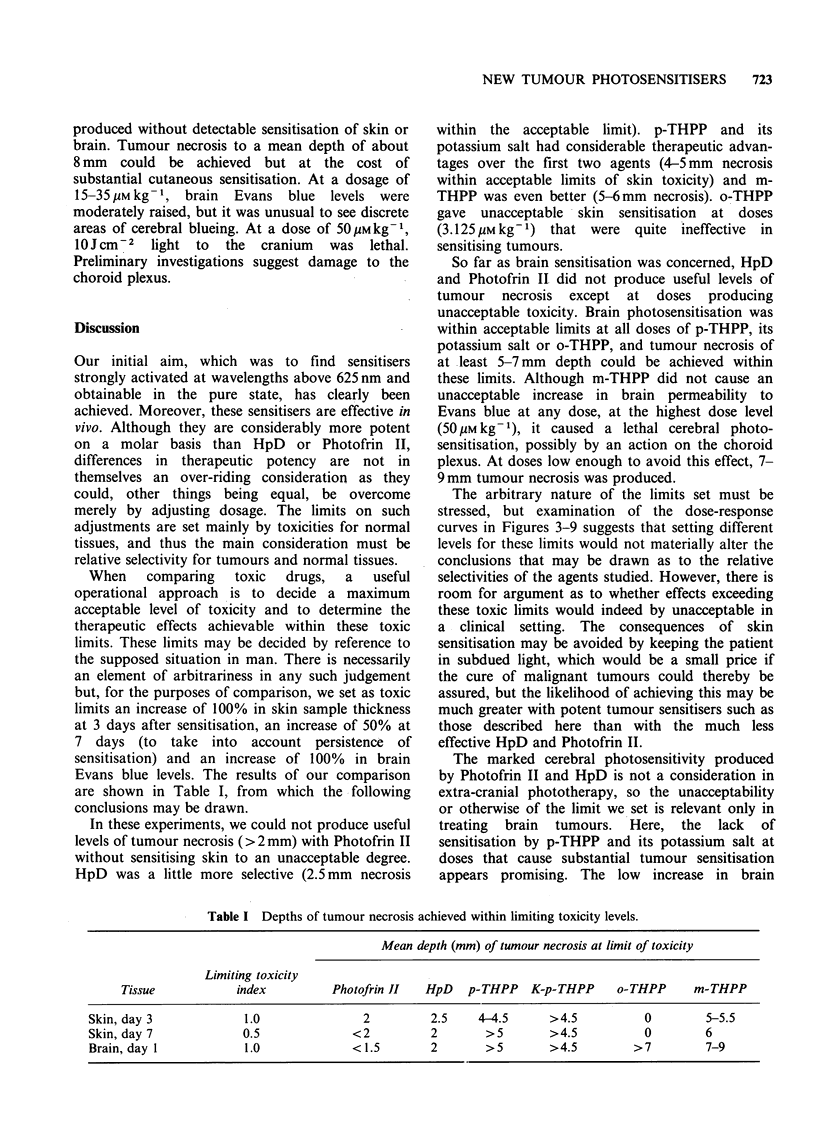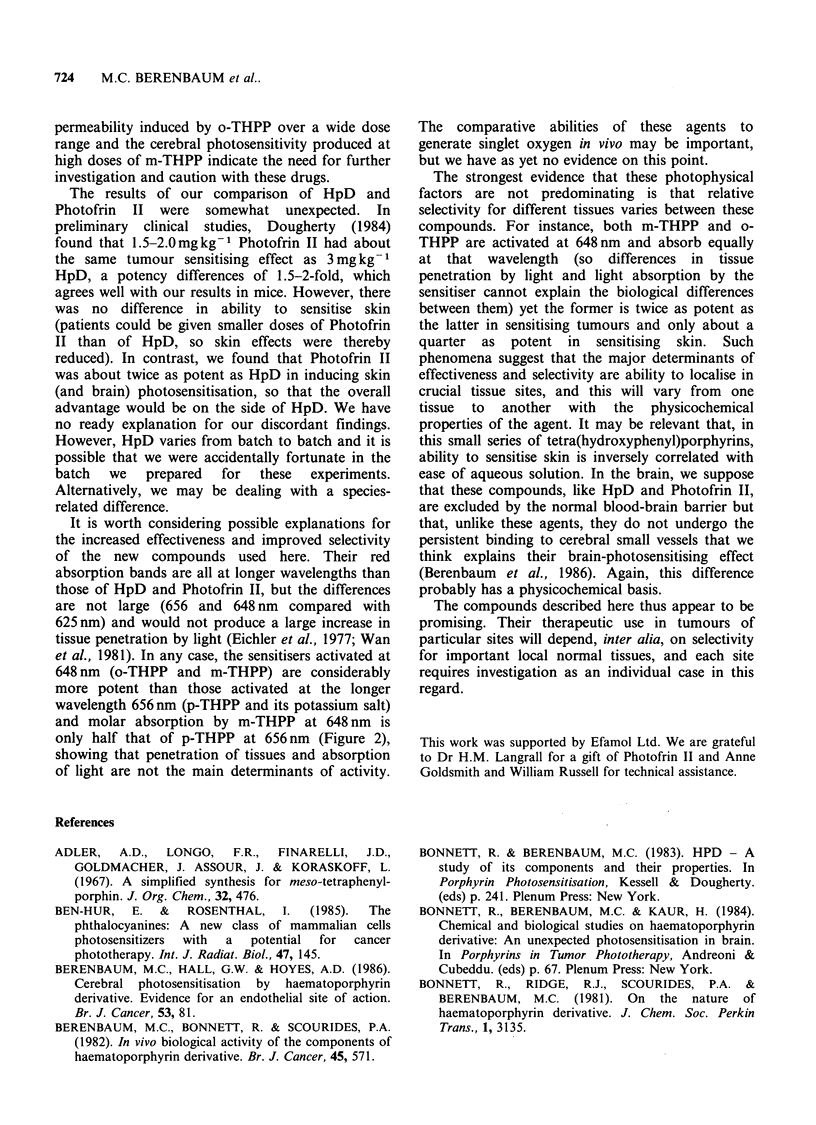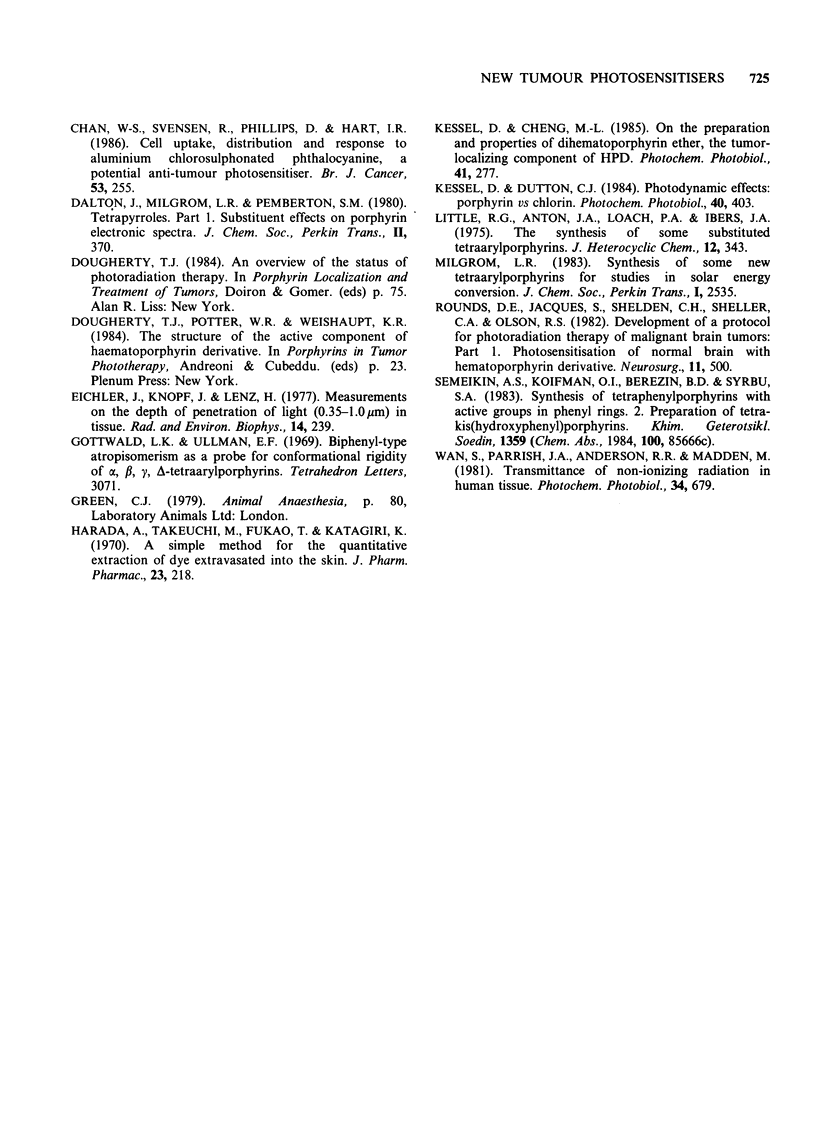# meso-Tetra(hydroxyphenyl)porphyrins, a new class of potent tumour photosensitisers with favourable selectivity.

**DOI:** 10.1038/bjc.1986.232

**Published:** 1986-11

**Authors:** M. C. Berenbaum, S. L. Akande, R. Bonnett, H. Kaur, S. Ioannou, R. D. White, U. J. Winfield

## Abstract

We compared para-, meta- and ortho-isomers of meso-tetra(hydroxyphenyl)porphyrin (p-, m- and o-THPP) and the potassium salt of the para compound (K-p-THPP) with haematoporphyrin derivative (HpD) and Photofrin II in their ability to sensitise tumours, skin and brain to light. HpD and Photofrin II induced modest tumour photosensitisation at the cost of substantial skin and brain sensitisation. At doses low enough to keep sensitisation of these normal tissues within acceptable limits, tumour sensitisation was sufficient to give necrosis only approximately 2 mm deep after exposure to 10 J cm-2 light. In contrast, doses of p-THPP, K-p-THPP and m-THPP that produced skin and brain sensitivity within acceptable limits sensitised tumours enough to give 4-9 mm necrosis after 10 J cm-2 light. m-THPP was, on a molar basis, about 25-30 times as potent as HpD and Photofrin II in sensitising tumours. o-THPP was also a potent tumour photosensitiser, but induced a prohibitive degree of skin photosensitivity even at low doses. It is unlikely that these differences in relative selectivity are due to differences in such photophysical parameters as optimum activating wavelength (which would affect tissue penetration by light), or light absorption, and physicochemical factors that determine tissue localisation may be involved. The high tumour sensitising potency and favourable tissue selectivity of m-THPP, p-THPP and K-p-THPP make them promising candidates for clinical tumour phototherapy.


Br. J. Cancer (1986) 54, 717–725

### *meso*-Tetra(hydroxyphenyl)porphyrins, a new class of potent tumour photosensitisers with favourable selectivity

M.C. Berenbaum[1], S.L. Akande[1], R. Bonnett[2], H. Kaur[2], S. Ioannou[2], R.D. White[2] & U.-J. Winfield[2]

[1]*Department of Experimental Pathology, St Mary's Hospital Medical School, London, W2 1PG and*
[2]*Department of Chemistry, Queen Mary College, London E1 4NS, UK.*

**Summary** We compared para-, meta- and ortho-isomers of *meso*-tetra(hydroxyphenyl)porphyrin (p-, m- and o-THPP) and the potassium salt of the para compound (K-p-THPP) with haematoporphyrin derivative (HpD) and Photofrin II in their ability to sensitise tumours, skin and brain to light. HpD and Photofrin II induced modest tumour photosensitisation at the cost of substantial skin and brain sensitisation. At doses low enough to keep sensitisation of these normal tissues within acceptable limits, tumour sensitisation was sufficient to give necrosis only ~2 mm deep after exposure to $10\,J\,cm^{-2}$ light.

In contrast, doses of p-THPP, K-p-THPP and m-THPP that produced skin and brain sensitivity within acceptable limits sensitised tumours enough to give 4–9 mm necrosis after $10\,J\,cm^{-2}$ light. m-THPP was, on a molar basis, about 25–30 times as potent as HpD and Photofrin II in sensitising tumours. o-THPP was also a potent tumour photosensitiser, but induced a prohibitive degree of skin photosensitivity even at low doses.

It is unlikely that these differences in relative selectivity are due to differences in such photophysical parameters as optimum activating wavelength (which would affect tissue penetration by light), or light absorption, and physicochemical factors that determine tissue localisation may be involved.

The high tumour sensitising potency and favourable tissue selectivity of m-THPP, p-THPP and K-p-THPP make them promising candidates for clinical tumour phototherapy.

'Haematoporphyrin derivative' (HpD) has for more than a decade been almost the sole agent used in clinical tumour phototherapy. However, it is well-known to have a number of drawbacks. First, it is a complex mixture, consisting in large part of materials inactive *in vivo* (Berenbaum *et al.*, 1982) and its composition varies between batches in a way that is not entirely controllable. Second, tissue penetration of light in the range able to activate photosensitisers increases with wavelength (Eichler *et al.*, 1977; Wan *et al.*, 1981), and thus, for effective tumour damage, illumination should be at the longest wavelength that excites the sensitiser. For HpD, this is around 620–630 nm, but is a poorly effective excitation band for this sensitiser.

Other disadvantages relate to tissue selectivity of photosensitisation. HpD sensitises skin, so that patients must avoid strong light for some weeks after treatment. HpD also sensitises other normal tissues, which compromises its use against tumours of certain sites, especially brain (Rounds *et al.*, 1982; Bonnett *et al.*, 1984; Berenbaum *et al.*, 1986).

We suggested (Bonnett & Berenbaum, 1983; Bonnett *et al.*, 1984; Berenbaum *et al.*, 1982) that the constituent of HpD responsible for tumour sensitisation was a dimer, possibly di-haematoporphyrin ether or ester. This idea has been endorsed by others (Dougherty, 1984*b*; Kessel & Cheng, 1985), but the evidence is incomplete. A partly purified preparation of HpD, said to be enriched in di-haematoporphyrin ether, has become available under the name Photofrin II. It is claimed that this preparation is a more potent tumour sensitiser than HpD and, in therapeutically equivalent dosage, has less skin sensitising activity (Dougherty, 1984). However, this material shares the main disadvantages of HpD, i.e., incompletely defined composition and weak excitation by red light.

It has long been apparent that it would be preferable to have photosensitisers that were pure materials, activated strongly by red light (and preferably at the more penetrating wavelengths above 620–630 nm). Favoured candidates are phthalocyanines (Ben-Hur & Rosenthal, 1985; Chan *et al.*, 1986) and chlorins (Kessel & Dutton, 1984). We have studied a wide range of porphyrins and related compounds. Included in these were a series of known *meso*-tetra(hydroxyphenyl)-porphyrins (Gottwald & Ullman, 1969; Little *et al.*, 1975; Semeikin *et al.*, 1983) which were selected since they, or their anions, were expected to show enhanced absorption in the red region (Milgrom, 1983). In the event, these compounds proved to be highly effective tumour photo-sensitisers with interesting and possibly useful tissue

Correspondence: M.C. Berenbaum.
Received 6 June 1986; and in revised form, 4 August 1986.

© The Macmillan Press Ltd., 1986

selectivity. We also synthesised a number of analogues of these substances. Preliminary results on these compounds were published in the patent literature (British Patent Application 84/29845, November 1984) and more extensive work on the hydroxyphenyl derivatives is now presented.

### Materials and methods

*Porphyrin photosensitisers*

The porphyrins used in the present work are known compounds which are available by the Rothemund synthesis (Adler *et al.*, 1967). We have improved the syntheses and purification procedures in various ways which will be described elsewhere. The structures, names and abbreviations are shown in Figure 1.

5, 10, 15, 20-Tetra(*p*-hydroxyphenyl)porphyrin (I) was prepared from p-acetoxy-benzaldehyde and pyrrole along the general route outlined by Little *et al.* (1975) but with considerable improvements. Mass spectrum (FAB): $M^+ + H$, 679 ($C_{44}H_{30}N_4O_4 + H$ requires 679). The potassium salt (II) was prepared as follows. The porphyrin (I) (29 mg) was dissolved in the minimum volume of tetrahydrofuran to which was added dropwise a concentrated solution of potassium methoxide (freshly prepared by dissolving potassium in anhydrous methanol) until the solution turned green and precipitate was formed. The precipitate was collected at the centrifuge, washed with a little tetra-hydrofuran and dried *in vacuo* to give the potassium salt of I as a dark purple solid (24 mg). This is regarded as the tetrapotassium tetraphenoxide (Milgrom, 1983). Attempts to purify this led to hydrolysis of the salt, and it was used without further purification.

5, 10, 15, 20-Tetra(*m*-hydroxyphenyl)porphyrin (III) was prepared from the corresponding tetramethyl ether (Dalton *et al.*, 1980) by demethylation with boron tribromide (Milgrom, 1983) rather than hydrogen bromide or aniline hydrochloride (Semeikin *et al.*, 1983). It was also prepared by the alkaline hydrolysis of *meso*-tetra(*m*-acetoxyphenyl)-porphyrin in an analogous way to the para compound (above). Mass spectrum (FAB): $M_+ + H$, 679.

5, 10, 15, 20-Tetra(*o*-hydroxyphenyl)porphyrin (IV) was prepared by ether cleavage ($BBr_3$) of the corresponding tetramethyl ether, and was obtained as a mixture of atropisomers (Gottwald & Ullman, 1969) which was used as such in the biological assay.

Porphyrins, I, III and IV were (apart from atropisomers in the last case) single substances on thin layer chromatography, and spectroscopic data (electronic and nuclear magnetic resonance spectra) confirmed the assigned structures.

HpD was prepared as previously described (Bonnett *et al.*, 1981). It was dissolved at a concentration of 4 mg ml$^{-1}$ in 0.5% sodium bicarbonate (Analar British Drug Houses) in pH 7.3 PBS. Photofrin II (2.5 mg ml$^{-1}$) was a generous gift of Photofrin Medical Inc.

*Solutions* All THPP's were soluble to some extent in dimethyl sulphoxide (DMSO) and aqueous alkali. However, o-THPP was so poorly soluble in aqueous media that only a DMSO solution was used (except for one experiment) and K-p-THPP was poorly soluble in DMSO so only aqueous solutions were used. p-THPP and K-p-THPP dissolved well in 0.0125 M sodium hydroxide in physiological saline, whereas m-THPP required 0.05 M sodium hydroxide for solution. For convenience, therefore, all aqueous solutions of THPP's were in 0.05 M sodium hydroxide in physiological saline. Stocks of solutions were made such that the required dose was given in 0.1 ml 10 g$^{-1}$ body weight of aqueous solution or 0.025 ml 10 g$^{-1}$ of DMSO. Solutions were stored at −20°C and thawed just before use. The chemical stability of these compounds in solution is under investigation. There was no detectable change in biological activity over the period of storage, which was generally a few weeks.

Drugs were administered on a mole/kg basis, with molecular weights of 680 for p-, m- and o-THPP, of 830 for K-p-THPP (Milgrom, 1983) and an assumed molecular weight of 600 for HpD and Photofrin II.

*Absorbances* 20 $\mu$M solutions of THPP's were prepared by injecting 40 $\mu$l of a 1.25 mM solution in DMSO into 2 ml of foetal calf serum (Flow Laboratories). Solutions of HpD and Photofrin II were similarly prepared in foetal calf serum). Absorbances were measured on a Beckman DU6 Spectrophotometer.

*Light source* An Oxford Lasers Cu10 copper-vapour laser, pumping a DL 10K dye laser was used. The dye was rhodamine 640. Light was passed down a 1 mm fibre and had a divergence of 30° at the fibre end. By using a mechanical attenuator in the beam or varying the distance between fibre tip and target, light intensity (measured with a 14BT thermopile (Laser Instrumentation)) was kept in the range 200–300 mW cm$^{-2}$, where thermal effects with red light are negligible. Excitation wavelengths were 625 nm for Photofrin II and HpD, 656 nm for p-THPP and K-p-THPP and 648 nm for m- and o-THPP.

I *para*-THPP

5,10,15,20-Tetra-

(*p*-hydroxyphenyl)porphyrin

III *meta*-THPP

5,10,15,20-Tetra-

(*m*-hydroxyphenyl)porphyrin

IV *ortho*-THPP

5,10,15,20-Tetra-

(*o*-hydroxyphenyl)porphyrin

**Figure 1** Structures, names and abbreviations of the three isomeric tetra(hydroxyphenyl)porphyrins used in this work.

*Animals*

Inbred BALB/c mice of either sex were used, weighing 16–22 g at the start of an experiment. All experiments on tumours (and tumour passage) were carried out on female mice. Experiments on skin were carried out in female mice (with one exception) and experiments on brain in male mice, but there were no evident sex-related differences in effect.

*Tumour necrosis*

The method for measuring treatment-induced tumour necrosis has been described in detail (Berenbaum *et al.*, 1982). Briefly, s.c. implants of the PC6 plasma cell tumour were illuminated with a light dose of $10\,J\,cm^{-2}$ at the appropriate wavelength one day after injection of sensitiser.

The next day, 0.2 ml of 1% Evans blue (Sigma) in saline was given i.v., tumours removed into formol saline 1 h later, and the depth of necrosis measured on slices of the fixed tumour using a dissecting microscope fitted with an eyepiece graticule.

*Skin damage*

The skin of sensitised mice rapidly becomes oedematous on exposure to light. This change was assessed by weighing a disc of skin 1 cm in diameter. Sensitised mice were shaved over the back 1–3 days before illumination and the area depilated with Immac (Anne French, London) immediately before illumination. Mice were anaesthetised with 'Equithesin' (Green, 1979) and a metal shield with a 1 cm diameter circular hole placed over the upper lumbar region. (It was important to avoid pressure on the skin by the shield as even this moderate restriction of blood supply during illumination substantially reduced damage). The skin exposed by the hole was illuminated to a dose of $10\,J\,cm^{-2}$ at the required wavelength. (Skin showing active hair growth was not used). Four hours later, mice were killed with ether. The dorsal skin was removed, placed dermis down on the absorbent side of a sheet of Benchkote (Gallenkemp Ltd), the illuminated area of skin, with supporting Benchkote, punched out with a 1 cm diameter stainless steel punch and rapidly weighed.

Control mice were either anaesthetised only or exposed to light without having been sensitised. The mean weight of the skin sample in these was $36 \pm 1$ mg ($n = 22$). Exposure of unsensitised mice to $100\,J\,cm^{-2}$ at any of the wavelengths used had no effect on skin weight.

*Brain damage*

The procedure has been described in detail previously (Berenbaum *et al.*, 1986). Briefly, mice were anaesthetised, the skin over the cranium was reflected, the cranium exposed to a dose of $10\,J\,cm^{-2}$ of light and the incision was then sutured. Three hours later, 0.2 ml of Evans blue in 10% bovine serum albumin was given i.v. and, 1 h later, mice were killed with ether and brains crudely homogenised in 20 ml Harada's solution (Harada *et al.*, 1970). The next day the suspension was centrifuged and the dye content of the supernate measured by absorbance at 620 nm. Mean Evans blue brain content in control mice subjected to anaesthesia and the surgical procedure only was $0.95 \pm 0.05\,\mu g$ ($n = 13$).

*Damage index*

To compare effects in the three tissues examined, a Damage Index was calculated. For tumours, this was simply the mean depth of tumour necrosis in *mm*. For skin and brain the index was calculated from mean skin sample weight or brain Evans blue content as follows:

$$\text{Damage Index} = (\text{Treated} - \text{Control})/\text{Control}$$

Thus, the index for undamaged tissues did not differ significantly from zero, and an index of 1 implied an increase of 100% in skin sample weight or brain Evans blue content.

*Sensitisation*

Solutions in 0.05 M sodium hydroxide/saline were given intravenously under brief ether anaesthesia in a volume of $0.1\,ml\ 10\,g^{-1}$. Solutions in DMSO were given i.p. generally in a volume of $0.025\,ml\ 10\,g^{-1}$. Injected mice were immediately placed in subdued light and kept there until they were due to receive laser treatement.

### Results

The absorption spectra in foetal calf serum of the agents used are shown in Figure 2. The wavebands selected for use were 625 nm for Photofrin II and HpD, 656 nm for p-THPP and its potassium salt and 648 nm for m-THPP and o-THPP.

Tumours could be sensitised by HpD sufficiently to show substantial necrosis (3–5 mm) on exposure to $10\,J\,cm^{-2}$ light but, at the large doses of sensitiser needed for this effect ($100–200\,\mu M\,kg^{-1}$), skin photosensitisation was marked (Figure 3). Even one week after injection of such doses of

**Figure 2** Absorption spectra of 25 μM solutions of (····) Photofrin II, (——) m-THPP, (-----) o-THPP and (-·-·-) p-THPP in foetal calf serum containg 2% (v/v) DMSO. The spectra of HpD and K-p-THPP are not shown as, over this range, they did not differ materially from those of Photofrin II and p-THPP respectively.

**Figure 3** Dose-response curves for HpD after exposure to 10 J cm$^{-2}$ light at 625 nm (pooled results of several experiments). Depth of tumour necrosis (mm) (■). Damage index for brain (●). Damage index for skin 3 days after sensitisation (▲). Damage index for skin 7 days after sensitisation (▼). (For definition of damage index, see text). Points show mean ± s.e. The number of samples per point for tumours is indicated by each point. For skin and brain, there were 5–7 samples per point.

HpD, exposure to this small dose of light caused an acute increase of 100–200% in skin sample weight. Cerebral photosensitisation was pronounced after 100 μM kg$^{-1}$ of HpD, with intense blueing of the brain surface.

**Figure 4** Dose-response curves for Photofrin II. Details as in **Figure 3**.

Photofrin II was about 1.5 times as potent as HpD in sensitising tumours, i.e., its dose-response curve was shifted to the left by a distance corresponding to a 1.5-fold reduction in dose (Figure 4). The shift in the curves for skin and brain sensitisation was greater – Photofrin II was about twice as potent as HpD in inducing skin oedema at 3 days and in increasing cerebral vessel permeability to Evans blue.

p-THPP and its potassium salt were 4–6 times as potent (on a μM kg$^{-1}$ basis) as Photofrin II and 5–6 times as potent as HpD in sensitising tumours. p-THPP was slightly more effective in sensitising tumours if given in DMSO i.p. rather than in aqueous i.v. In contrast to HpD and Photofrin II, p-THPP produced little sensitisation of skin at 3 days after injection except at high doses, and this had vanished at 7 days. Substantial brain sensitisation was not produced at any dose (Figure 5). The potassium salt of p-THPP behaved similarly

**Figure 5** Dose-response curves for p-THPP after exposure to 10 J cm$^{-2}$ light at 656 nm. Depth of tumour necrosis (in mm) after injection in aqueous alkali i.v. (■). Depth of tumour necrosis after injection in DMSO i.p. (□). Other details as in **Figure 3**.

(Figure 6), with the difference that virtually no skin sensitisation was present even at 3 days after injection.

o-THPP was about 12–16 times as potent as HpD and tumour destruction to a mean depth of 7 mm was achieved (Figure 7), but this was at the cost of intense skin sensitisation. Even with doses as low as 6.25 $\mu$M kg$^{-1}$ (which produced $\sim$3.5 mm tumour necrosis), samples from skin illuminated 3 or 7 days later were four times the normal weight. Evans blue injection showed that, at doses of 6.25 $\mu$M kg$^{-1}$ or above of o-THPP, skin exposed to 10 J cm$^{-2}$ light 3 days later rapidly became avascular (and the same happened on exposure at 7 days after 12.5–25 $\mu$M kg$^{-1}$). At these dose levels, skin oedema was maximal and, at higher doses still (50 $\mu$M kg$^{-1}$), it was reduced, presumably because vascular damage was so marked as to compromise blood flow to the illuminated area and its surroundings.

The possibility that the intense cutaneous photosensitisation induced by o-THPP was associated with administration in DMSO was tested by comparing the effects of low doses in both DMSO and aqueous alkali (Figure 8). No differences were apparent.

In brains illuminated after injecting o-THPP, there was a slight increase in Evans blue levels over a wide dose-range, but rarely discrete areas of blueing of the cerebral surface.

m-THPP was the most potent tumour photosensitiser in this series (Figure 9), being about 25–30 times as potent as HpD. Solutions in aqueous alkali and DMSO were equally effective. At doses of 6.25 $\mu$M kg$^{-1}$, 4–5 mm tumour necrosis was

**Figure 6** Dose-response curves for K-p-THPP in aqueous alkali i.v. after exposure to 10 J cm$^{-2}$ light at 656 nm. Details as in **Figure 3**.

**Figure 7** Dose-response curves for o-THPP in DMSO i.p. after exposure to 10 J cm$^{-2}$ light at 648 nm. Details as in **Figure 3**.

**Figure 8** Dose-response curves for skin oedema after sensitisation with o-THPP in aqueous alkali i.v. (▼) or in DMSO i.p. (▽). Skin in male mice exposed to 10 J cm$^{-2}$ light at 648 nm 7 days after injection of sensitiser. Groups of 5.

**Figure 9** Dose-response curves for m-THPP after exposure to 10 J cm$^{-2}$ light at 648 nm. Details as in **Figure 5**.

produced without detectable sensitisation of skin or brain. Tumour necrosis to a mean depth of about 8 mm could be achieved but at the cost of substantial cutaneous sensitisation. At a dosage of 15–35 $\mu$M kg$^{-1}$, brain Evans blue levels were moderately raised, but it was unusual to see discrete areas of cerebral blueing. At a dose of 50 $\mu$M kg$^{-1}$, 10 J cm$^{-2}$ light to the cranium was lethal. Preliminary investigations suggest damage to the choroid plexus.

### Discussion

Our initial aim, which was to find sensitisers strongly activated at wavelengths above 625 nm and obtainable in the pure state, has clearly been achieved. Moreover, these sensitisers are effective *in vivo*. Although they are considerably more potent on a molar basis than HpD or Photofrin II, differences in therapeutic potency are not in themselves an over-riding consideration as they could, other things being equal, be overcome merely by adjusting dosage. The limits on such adjustments are set mainly by toxicities for normal tissues, and thus the main consideration must be relative selectivity for tumours and normal tissues.

When comparing toxic drugs, a useful operational approach is to decide a maximum acceptable level of toxicity and to determine the therapeutic effects achievable within these toxic limits. These limits may be decided by reference to the supposed situation in man. There is necessarily an element of arbitrariness in any such judgement but, for the purposes of comparison, we set as toxic limits an increase of 100% in skin sample thickness at 3 days after sensitisation, an increase of 50% at 7 days (to take into account persistence of sensitisation) and an increase of 100% in brain Evans blue levels. The results of our comparison are shown in Table I, from which the following conclusions may be drawn.

In these experiments, we could not produce useful levels of tumour necrosis (>2 mm) with Photofrin II without sensitising skin to an unacceptable degree. HpD was a little more selective (2.5 mm necrosis within the acceptable limit). p-THPP and its potassium salt had considerable therapeutic advantages over the first two agents (4–5 mm necrosis within acceptable limits of skin toxicity) and m-THPP was even better (5–6 mm necrosis). o-THPP gave unacceptable skin sensitisation at doses (3.125 $\mu$M kg$^{-1}$) that were quite ineffective in sensitising tumours.

So far as brain sensitisation was concerned, HpD and Photofrin II did not produce useful levels of tumour necrosis except at doses producing unacceptable toxicity. Brain photosensitisation was within acceptable limits at all doses of p-THPP, its potassium salt or o-THPP, and tumour necrosis of at least 5–7 mm depth could be achieved within these limits. Although m-THPP did not cause an unacceptable increase in brain permeability to Evans blue at any dose, at the highest dose level (50 $\mu$M kg$^{-1}$), it caused a lethal cerebral photosensitisation, possibly by an action on the choroid plexus. At doses low enough to avoid this effect, 7–9 mm tumour necrosis was produced.

The arbitrary nature of the limits set must be stressed, but examination of the dose-response curves in Figures 3–9 suggests that setting different levels for these limits would not materially alter the conclusions that may be drawn as to the relative selectivities of the agents studied. However, there is room for argument as to whether effects exceeding these toxic limits would indeed by unacceptable in a clinical setting. The consequences of skin sensitisation may be avoided by keeping the patient in subdued light, which would be a small price if the cure of malignant tumours could thereby be assured, but the likelihood of achieving this may be much greater with potent tumour sensitisers such as those described here than with the much less effective HpD and Photofrin II.

The marked cerebral photosensitivity produced by Photofrin II and HpD is not a consideration in extra-cranial phototherapy, so the unacceptability or otherwise of the limit we set is relevant only in treating brain tumours. Here, the lack of sensitisation by p-THPP and its potassium salt at doses that cause substantial tumour sensitisation appears promising. The low increase in brain

**Table I** Depths of tumour necrosis achieved within limiting toxicity levels.

| | | *Mean depth (mm) of tumour necrosis at limit of toxicity* | | | | | |
|---|---|---|---|---|---|---|---|
| *Tissue* | *Limiting toxicity index* | *Photofrin II* | *HpD* | *p-THPP* | *K-p-THPP* | *o-THPP* | *m-THPP* |
| Skin, day 3 | 1.0 | 2 | 2.5 | 4–4.5 | >4.5 | 0 | 5–5.5 |
| Skin, day 7 | 0.5 | <2 | 2 | >5 | >4.5 | 0 | 6 |
| Brain, day 1 | 1.0 | <1.5 | 2 | >5 | >4.5 | >7 | 7–9 |

permeability induced by o-THPP over a wide dose range and the cerebral photosensitivity produced at high doses of m-THPP indicate the need for further investigation and caution with these drugs.

The results of our comparison of HpD and Photofrin II were somewhat unexpected. In preliminary clinical studies, Dougherty (1984) found that 1.5–2.0 $mg\,kg^{-1}$ Photofrin II had about the same tumour sensitising effect as 3 $mg\,kg^{-1}$ HpD, a potency differences of 1.5–2-fold, which agrees well with our results in mice. However, there was no difference in ability to sensitise skin (patients could be given smaller doses of Photofrin II than of HpD, so skin effects were thereby reduced). In contrast, we found that Photofrin II was about twice as potent as HpD in inducing skin (and brain) photosensitisation, so that the overall advantage would be on the side of HpD. We have no ready explanation for our discordant findings. However, HpD varies from batch to batch and it is possible that we were accidentally fortunate in the batch we prepared for these experiments. Alternatively, we may be dealing with a species-related difference.

It is worth considering possible explanations for the increased effectiveness and improved selectivity of the new compounds used here. Their red absorption bands are all at longer wavelengths than those of HpD and Photofrin II, but the differences are not large (656 and 648 nm compared with 625 nm) and would not produce a large increase in tissue penetration by light (Eichler *et al.*, 1977; Wan *et al.*, 1981). In any case, the sensitisers activated at 648 nm (o-THPP and m-THPP) are considerably more potent than those activated at the longer wavelength 656 nm (p-THPP and its potassium salt) and molar absorption by m-THPP at 648 nm is only half that of p-THPP at 656 nm (Figure 2), showing that penetration of tissues and absorption of light are not the main determinants of activity. The comparative abilities of these agents to generate singlet oxygen *in vivo* may be important, but we have as yet no evidence on this point.

The strongest evidence that these photophysical factors are not predominating is that relative selectivity for different tissues varies between these compounds. For instance, both m-THPP and o-THPP are activated at 648 nm and absorb equally at that wavelength (so differences in tissue penetration by light and light absorption by the sensitiser cannot explain the biological differences between them) yet the former is twice as potent as the latter in sensitising tumours and only about a quarter as potent in sensitising skin. Such phenomena suggest that the major determinants of effectiveness and selectivity are ability to localise in crucial tissue sites, and this will vary from one tissue to another with the physicochemical properties of the agent. It may be relevant that, in this small series of tetra(hydroxyphenyl)porphyrins, ability to sensitise skin is inversely correlated with ease of aqueous solution. In the brain, we suppose that these compounds, like HpD and Photofrin II, are excluded by the normal blood-brain barrier but that, unlike these agents, they do not undergo the persistent binding to cerebral small vessels that we think explains their brain-photosensitising effect (Berenbaum *et al.*, 1986). Again, this difference probably has a physicochemical basis.

The compounds described here thus appear to be promising. Their therapeutic use in tumours of particular sites will depend, *inter alia*, on selectivity for important local normal tissues, and each site requires investigation as an individual case in this regard.

This work was supported by Efamol Ltd. We are grateful to Dr H.M. Langrall for a gift of Photofrin II and Anne Goldsmith and William Russell for technical assistance.

### References

ADLER, A.D., LONGO, F.R., FINARELLI, J.D., GOLDMACHER, J. ASSOUR, J. & KORASKOFF, L. (1967). A simplified synthesis for *meso*-tetraphenylporphin. *J. Org. Chem.*, **32**, 476.

BEN-HUR, E. & ROSENTHAL, I. (1985). The phthalocyanines: A new class of mammalian cells photosensitizers with a potential for cancer phototherapy. *Int. J. Radiat. Biol.*, **47**, 145.

BERENBAUM, M.C., HALL, G.W. & HOYES, A.D. (1986). Cerebral photosensitisation by haematoporphyrin derivative. Evidence for an endothelial site of action. *Br. J. Cancer*, **53**, 81.

BERENBAUM, M.C., BONNETT, R. & SCOURIDES, P.A. (1982). *In vivo* biological activity of the components of haematoporphyrin derivative. *Br. J. Cancer*, **45**, 571.

BONNETT, R. & BERENBAUM, M.C. (1983). HPD – A study of its components and their properties. In *Porphyrin Photosensitisation*, Kessell & Dougherty. (eds) p. 241. Plenum Press: New York.

BONNETT, R., BERENBAUM, M.C. & KAUR, H. (1984). Chemical and biological studies on haematoporphyrin derivative: An unexpected photosensitisation in brain. In *Porphyrins in Tumor Phototherapy*, Andreoni & Cubeddu. (eds) p. 67. Plenum Press: New York.

BONNETT, R., RIDGE, R.J., SCOURIDES, P.A. & BERENBAUM, M.C. (1981). On the nature of haematoporphyrin derivative. *J. Chem. Soc. Perkin Trans.*, **1**, 3135.

CHAN, W-S., SVENSEN, R., PHILLIPS, D. & HART, I.R. (1986). Cell uptake, distribution and response to aluminium chlorosulphonated phthalocyanine, a potential anti-tumour photosensitiser. *Br. J. Cancer,* **53,** 255.

DALTON, J., MILGROM, L.R. & PEMBERTON, S.M. (1980). Tetrapyrroles. Part 1. Substituent effects on porphyrin electronic spectra. *J. Chem. Soc., Perkin Trans.,* **II,** 370.

DOUGHERTY, T.J. (1984). An overview of the status of photoradiation therapy. In *Porphyrin Localization and Treatment of Tumors,* Doiron & Gomer. (eds) p. 75. Alan R. Liss: New York.

DOUGHERTY, T.J., POTTER, W.R. & WEISHAUPT, K.R. (1984). The structure of the active component of haematoporphyrin derivative. In *Porphyrins in Tumor Phototherapy,* Andreoni & Cubeddu. (eds) p. 23. Plenum Press: New York.

EICHLER, J., KNOPF, J. & LENZ, H. (1977). Measurements on the depth of penetration of light (0.35–1.0 $\mu$m) in tissue. *Rad. and Environ. Biophys.,* **14,** 239.

GOTTWALD, L.K. & ULLMAN, E.F. (1969). Biphenyl-type atropisomerism as a probe for conformational rigidity of $\alpha$, $\beta$, $\gamma$, $\Delta$-tetraarylporphyrins. *Tetrahedron Letters,* 3071.

GREEN, C.J. (1979). *Animal Anaesthesia,* p. 80, Laboratory Animals Ltd: London.

HARADA, A., TAKEUCHI, M., FUKAO, T. & KATAGIRI, K. (1970). A simple method for the quantitative extraction of dye extravasated into the skin. *J. Pharm. Pharmac.,* **23,** 218.

KESSEL, D. & CHENG, M.-L. (1985). On the preparation and properties of dihematoporphyrin ether, the tumor-localizing component of HPD. *Photochem. Photobiol.,* **41,** 277.

KESSEL, D. & DUTTON, C.J. (1984). Photodynamic effects: porphyrin *vs* chlorin. *Photochem. Photobiol.,* **40,** 403.

LITTLE, R.G., ANTON, J.A., LOACH, P.A. & IBERS, J.A. (1975). The synthesis of some substituted tetraarylporphyrins. *J. Heterocyclic Chem.,* **12,** 343.

MILGROM, L.R. (1983). Synthesis of some new tetraarylporphyrins for studies in solar energy conversion. *J. Chem. Soc., Perkin Trans.,* **I,** 2535.

ROUNDS, D.E., JACQUES, S., SHELDEN, C.H., SHELLER, C.A. & OLSON, R.S. (1982). Development of a protocol for photoradiation therapy of malignant brain tumors: Part 1. Photosensitisation of normal brain with hematoporphyrin derivative. *Neurosurg.,* **11,** 500.

SEMEIKIN, A.S., KOIFMAN, O.I., BEREZIN, B.D. & SYRBU, S.A. (1983). Synthesis of tetraphenylporphyrins with active groups in phenyl rings. 2. Preparation of tetrakis(hydroxyphenyl)porphyrins. *Khim. Geterotsikl. Soedin,* **1359** (*Chem. Abs.,* 1984, **100,** 85666c).

WAN, S., PARRISH, J.A., ANDERSON, R.R. & MADDEN, M. (1981). Transmittance of non-ionizing radiation in human tissue. *Photochem. Photobiol.,* **34,** 679.